# The need for active and integrated involvement of the community and health professionals in the prevention and control of dengue hemorrhagic fever in Indonesia

**DOI:** 10.11604/pamj.2024.47.185.43298

**Published:** 2024-04-16

**Authors:** Muhammad Farid Dimjati Lusno, Setya Haksama, Ririh Yudhastuti, Siti Zubaidah, Abdullah Al Mamun, Abubakar Tarawally, Muhammad Rifqo Hafidzudin Farid, Heru Santoso Wahito Nugroho

**Affiliations:** 1 Faculty of Public Health, Universitas Airlangga, Surabaya, Indonesia,; 2 Faculty of Medicine, Universitas Airlangga, Surabaya, Indonesia,; 3 Center of Excellence of Community Empowerment in Health, Poltekkes Kemenkes Surabaya, Surabaya, Indonesia

**Keywords:** Dengue fever management, vector control, proactive surveillance, public health strategy

## Abstract

Successful control and prevention of dengue fever requires active involvement from all parties. For this reason, three innovative programs are needed, namely: i) increasing knowledge, attitude and practice (KAP) of the community and health professionals as capital in controlling dengue fever in a sustainable manner; ii) application of “3M Plus” to suppress vector breeding in household settings; iii) promotion of the “Jumantik” program as an effective community empowerment approach to prevent and control dengue fever based on community independence. It was concluded that successful control of dengue fever requires integration of the community and health workers through various innovative programs.

## Opinion

In Indonesia, dengue hemorrhagic fever (DHF) is still a public health problem today [1], so of course strategic efforts are needed to solve it, and the best solution strategy is through programs that actively involve all parties, both health professionals and the community itself [2]. However, until now, the integration of various parties in preventing and controlling DHF has not worked well. For this reason, it is necessary to identify various programs that are already running and strengthen them with maximum integration of all relevant parties. The first is optimizing knowledge, attitudes and practices (KAP) regarding DHF control as initial capital to be able to control this disease in a sustainable manner. Community KAP needs to be compiled into a community participation index, so that it can be evaluated carefully on an ongoing basis. Apart from community KAP, the same thing must also be done for health professionals, so it is also necessary to optimize health professional KAP, as a companion to the community in controlling DHF [3]. Second is the optimization of the “3M plus” program (In Indonesian, 3M = *menguras, menutup, mendaurulang*; in English = drain, close and recycle). So, 3M is: i) draining water reservoirs, ii) closing water reservoirs, and; iii) recycling various items that have the potential to become mosquito breeding grounds. Meanwhile, the “Plus” referred to in this case includes: i) planting plants that can ward off mosquitoes; ii) checking places used for water storage; iii) keeping fish that eat mosquito larvae; iv) using mosquito repellent; v) install wire mesh on windows and ventilation in the house; vi) work together to clean the environment; vii) put used clothes in a closed container; vii) provide larvicide to water reservoirs that are difficult to drain; ix) repair channels and gutters that are not running smoothly [4,5].

It is important to realize that the active involvement of the entire community through the “3M Plus” program is very strategic, because it is the local community who can closely monitor the environmental conditions in which they live. In this way, they become the spearheads of environmental management, especially the eradication of mosquito nests, starting from the household [5]. Third is the mobilization of public health cadres in the prevention and control of DHF through the *"Jumantik*= larva monitors" program. Jumantik is the pioneer of DHF control in Indonesia, who has become a leader for other community members in fighting DHF which is a problem to this day. The details of jumantik's duties are: i) planning home visits in his area; ii) educate the public and eradicate larvae; iii) mobilize and supervise the community in controlling DHF; iv) record and summarize the results of larval examination; v) report the results of the larval examination to the community health center; vi) together with the supervisor, carry out monitoring of the local area and mapping of larval inspection results [6]. Controlling DHF is not easy [7,8]. All potential must be optimized to get the expected achievements. As a key to success, the three programs that have been launched by the Indonesian government should be implemented with harmonious and complete integration from both the community and health professionals as supervisors. Thus, it can be concluded that active and integrated involvement of the community and health professionals in various existing programs is a condition for the success of sustainable DHF control in Indonesia.
